# Bulk and Compound-Specific Stable Isotope Analysis for the Authentication of Walnuts (*Juglans regia*) Origins

**DOI:** 10.1021/acs.jafc.3c03770

**Published:** 2023-11-02

**Authors:** Zatil A. Athaillah, Chris Yarnes, Selina C. Wang

**Affiliations:** † Food Science and Technology Department of University of California, Davis, One Shields Avenue, Davis, California 95616, United States; ‡ Stable Isotope Facility of University of California, Davis, Davis, California 95616, United States

**Keywords:** walnut, stable isotope, bulk, compound-specific, origin authentication

## Abstract

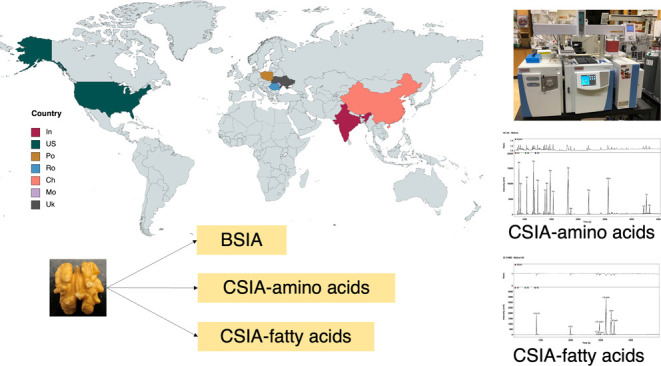

Walnuts are grown in various countries, and as product origin information is becoming more important to consumers, new techniques to differentiate walnut geographical authenticity are needed. We conducted bulk stable isotope analysis (BSIA) and compound-specific stable isotope analysis (CSIA) on walnuts grown in seven countries. The BSIA consisted of δ^13^C_bulk_, δ^15^N_bulk_, and δ^34^S_bulk_, and CSIA covered δ^2^H_fatty acid_, δ^13^C_fatty acid_, δ^13^C_amino acid_, δ^15^N_amino acid_, and δ^2^H_amino acid_. Analysis of variance (ANOVA) and linear discriminant analysis (LDA) were used for statistical analysis to compare samples from the USA and China. Parameters that yielded significant variations are δ^2^H_C18:1*n*–9_, δ^13^C_C18:2*n*–6_, δ^13^C_C18:3*n*–3_, δ^13^C_Gly_, δ^13^C_Leu_, δ^13^C_Val_, δ^2^H_Glu_, δ^2^H_Ile_, δ^2^H_Leu_, and δ^2^H_Thr_. Our findings suggested that CSIA of fatty acids and amino acids can be useful to differentiate the geographical provenance of walnuts.

## Introduction

Walnuts are known to have many health-promoting properties, namely, fatty acids, including high concentrations of linoleic acid, tocopherols, ellagitannins, and urolithins. Walnuts help protect against several neurological disorders.^1^ Walnut intake is also associated with reduced breast tumor incidence, multiplicity, and size;^2^ improved diastolic functions and cardiovascular risk factors;^3^ and its oil can lower cholesterol production, increase cholesterol efflux, and reduce total cholesterol and triacylglycerols in HepG2 cells.^4^ Walnut consumption has been shown to increase gut microbiome diversity as well.^5^ A study indicated that walnut oils may be useful as a plant-based infant formula ingredient because of the similar major fatty acids as human milk.^6^

Walnuts are grown in various countries including China, the USA, Chile, Iran, Ukraine, Moldova, Poland, India, and Romania. Similar to many other crops and food products, such as wines and olive oil, geographical origin is often linked with specific quality perception and consumers regard some countries as producing better quality products than the others.^7−9^ This can lead to economically motivated adulteration (EMA) by intentional origin mislabeling to increase profits. Currently, origin information is obtained from suppliers through simple documentation; therefore, its falsification is uncomplicated.

Stable isotope ratio measurements have been used to accurately differentiate geographical origin or agricultural practices associated with food commodities.^10^ For example, variation in the nitrogen stable isotope ratio has been associated with the types of fertilizer used (organic and synthetic). Plants cultivated using conventional methods generally have lower δ^15^N values than their organic counterparts^11,12^ because synthetic fertilizer used in conventional farms did not undergo isotope fractionation, relative to the reference standard which is atmospheric nitrogen.^13^ Among organically grown vegetables, those received animal manure have higher δ^15^N than those given green manure or catch crops^12^ because the increased ^15^N enrichment accompanying higher trophic level of animals, compared to plants.^14^

Isotope fractionation of hydrogen has been linked to evaporation, condensation, and precipitation^15^ that are specific to regions of cultivation. For example, δ^2^H values exhibit a correlation with the distance of growing location to coast. Precipitation that occurs at an increasing distance from the coast toward the inner continent is more depleted in δ^2^H, due to isotope fractionation through Rayleigh distillation processes (“continental” effect) as water vapor is transported away from the oceans.^16^ In addition, δ^2^H has been used to indicate milk that had been diluted with local water,^17^ as well as to distinguish the geographical provenance of sake^18^ and milk.^19^

Variation in carbon isotope ratios is significantly affected by photosynthetic types (C3 or C4)^15^ through additional environmental (e.g., elevation) and physiological variables (e.g., water use efficiency). Similar to nitrogen and hydrogen stable isotope ratios, there have also been several reports linking variation in carbon isotope ratios to geographical provenance.^20^ Carbon stable isotope of fatty acids could also identify maize oil that had been adulterated with oil from some C3 plants because of the difference mechanism in CO_2_ fixation.^21^

Stable isotope of sulfur can help identify pollution from mining activities.^22,23^ Plants exposed to acid mine water had depleted ^34^S (negative values), compared to nonaffected plants that had positive ^34^S values because sulfur from the mining area had minimum isotopic fractionation.^23^

Stable isotope analysis can be divided into bulk stable isotope analysis (BSIA) and compound-specific stable isotope analysis (CSIA). BSIA is conducted on whole samples; meanwhile, CSIA is done on a specific group of compounds extracted from the samples. BSIA is done using an elemental analyzer connected with isotope ratio mass spectrometry (EA-IRMS). Whole tissues of samples undergo either combustion (i.e., for detection of C and N) or thermal conversion (i.e., for detection of H) to convert the small elements of interest to gases. The gases are then ionized and detected using a sector field mass spectrometer.^24^

In this study, CSIA was performed on both fatty acids and amino acids. Multielement CSIA of fatty acids and amino acids has been associated with numerous environmental factors relevant to geographical provenance. Carbon and hydrogen stable isotopes of fatty acids have been known to reflect the geographical properties (e.g., elevation and precipitation, respectively), while stable isotopes of carbon and nitrogen of amino acids correspond to fertilizer and pesticide use. Hydrogen stable isotopes of amino acids and fatty acids have been linked with the source of water used by plants or animals.

While BSIA has been widely used for diverse purposes and requires simple sample preparation and less cost, some studies show that it may be less useful than CSIA, e.g., in differentiating organic from conventional shiitake mushrooms^25^ and in tracing the geographical origin of wines.^26^ CSIA may provide more detailed isotopic information than BSIA because BSIA data represent a mass balance of molecular constituents which results in information from the isotope fractionation of the individual components to be diluted or masked by co-occurring molecules.

In this study, we applied both BSIA and CSIA to compare their effectiveness in distinguishing walnut origins. BSIA was performed on three elements (carbon, sulfur, and nitrogen), while CSIA was measured on fatty acids (hydrogen and carbon) and amino acids (carbon, nitrogen, and hydrogen). This is the first comprehensive stable isotope study on walnuts, with regard to extensive parameters and sample origins. A previous study using stable isotopes δ^13^C_bulk_, δ^15^N_bulk_, and δ^2^H_bulk_ was done on kernels from Germany and France,^27^ and another one using δ^13^C_fatty acid_ on walnut oil from China.^28^

To process our data, analysis of variance (ANOVA) was applied to see if the parameters significantly differ across the regions, while multivariate analysis was applied to reduce data dimensionality and evaluate whether the samples can be classified based on the origins and which parameter, or combination of parameters, generates the most discrimination. An emphasis on comparing samples from China and the USA was performed because these two countries are the major walnut producers.

## Materials and Methods

### Sample

Walnuts (±2 kg) from seven countries: the USA (29 samples), China (5 samples), Poland (2 samples), Moldova (2 samples), India (1 sample), Romania (1 sample), and Ukraine (1 sample) were collected between July and September 2020. The USA samples were all from California and consisted of three cultivars commonly grown in the region: Chandler (9 samples), Howard (10 samples), and Tulare (10 samples). The China samples were sourced from Xinjiang (2 samples), Shaanxi (1 sample), Yunnan (1 sample), and an unknown province (1 sample). We did not have information on which province/state the remaining samples were grown. Each sample represented different suppliers or sources. For the USA, it also reflected different combinations of cultivars and growers.

For bulk and amino acid specific analyses, five kernel halves were peeled to remove the skin (pellicle) manually with a knife, then ground, and later dried at 45 °C to remove water (initial moisture content was ±4%, w/w). For fatty acid analysis, oil was extracted from each sample using an expeller (KK Oil Prince F Universal, Reut, Germany). Approximately 500 g of walnut kernels was fed into the expeller. A screw was rotated at 45 rpm. Walnut slurry containing oil came out from a perforated tube because of pressing action. Walnut cake, the byproduct, was extruded from die #10. The slurry was centrifuged at 10,000*g* for 15 min to separate walnut oil that was later used for CSIA-fatty acids.

### BSIA—δ^13^C, δ^15^N, and δ^34^S

Samples were analyzed on an elemental analyzer-isotope ratio mass spectrometer (EA-IRMS) for δ^13^C and δ^15^N, following a published study.^29^ Samples were combusted at 950 °C in a reactor packed with chromium oxide and silvered copper oxide. Oxygen was dosed during the sample introduction for complete combustion. Following complete combustion, residual oxygen and nitrogen oxides were removed by passing the combustion products over reduced copper at 650 °C. CO_2_ and N_2_ were separated by an adsorption trap in the Elementar EA (Elementar vario EL cube EA). After separation, an aliquot of the analyte gases was carried out to the IRMS, an Elementar VisION IRMS (Elementar Analysensysteme GmbH, Langenselbold, Germany), for measurement.

At least six laboratory reference materials were interspersed with samples and used for finalizing sample data and assessing the analytical quality. All of the laboratory references were calibrated against international reference materials. Final delta (δ) values were expressed relative to international standards Vienna Pee Dee Belemnite (VPDB) for carbon and atmospheric nitrogen (Air) for nitrogen, respectively. The mean measurement error of replicates was ±0.14 and ±0.07 for δ^13^C and δ^15^N, respectively, while measurement accuracy, as determined by quality control materials, was within ±0.07 and ±0.04 for δ^13^C and δ^15^N, respectively.

Samples were analyzed for δ^34^S following an earlier study^29^ on an Elementar Vario ISOTOPE cube elemental analyzer (Elementar Analysensysteme GmbH, Langenselbold, Germany) interfaced to an Elementar PrecisION isotope ratio mass spectrometer (Cheadle Hulme, Cheadle, England). Samples were combusted at 1150 °C in a reactor packed with tungsten oxide. Immediately following combustion, sample gases were reduced with elemental copper at 880 °C and subsequently passed through a buffering reactor filled with quartz chips held at 900 °C. SO_2_ and CO_2_ were then separated by adsorption columns, allowing for full separation and peak focusing. Following separation, the SO_2_ adsorption trap was heated and the sample SO_2_ was passed directly to the IRMS for measurement.

Multiple instances of at least four laboratory reference materials were interspersed with samples and used for the finalization of sample data and assessment of analytical quality. The mean measurement error of δ^34^S was ±0.21, while the measurement accuracy, as determined by quality control materials, was within ±0.07.

### CSIA—δ^2^H and δ^13^C of Fatty Acids

Sample preparation and analytical procedure followed some studies.^30,31^ Fatty acids in walnut oil were subjected to a derivatization step to generate fatty acid methyl esters (FAMEs). The FAMEs were dissolved in heptane and injected at 290 °C (splitless, 1 min). The separation was performed on an Agilent DB-5 ms Ultra Inert column (60 m × 0.25 mm ID × 1 μm film thickness) at a constant flow rate of 1.2 mL/min. The column temperature program was 80 °C (hold 1 min), 220 °C (4 °C/min), and 290 °C (10 °C/min; hold 30 min).

Gas chromatography–combustion–pyrolysis (GC–C–P-IRMS) was performed on a Thermo Trace GC 1310 gas chromatograph connected to a Thermo Finnigan MAT 253 isotope ratio mass spectrometer via a GC IsoLink II combustion interface. For ^13^C analysis, individual FAMEs were converted to CO_2_ within a combustion reactor composed of a NiO tube containing CuO and NiO wires maintained at 1000 °C. Water was subsequently removed using a Nafion dryer before the analyte gases were transferred to the IRMS. For ^2^H analysis, individual FAMEs were converted to H_2_ within a high-temperature thermal conversion reactor of a graphitized Al_2_O_3_ tube maintained at 1425 °C.

One of every eight samples was analyzed in duplicate; further replicates were analyzed if initial measurements fell outside the expected measurement error. Replicates of the quality control and assurance reference materials were measured in every eight samples.

Quality control and assurance mixtures were composed of pure FAMEs (and FAs) that had been calibrated separately by EA- and high-temperature conversion (TC)/EA-IRMS using certified reference materials (e.g., NBS-22 and IAEA-CH-7) distributed by the United States Geological Survey (USGS), the National Institute of Standards and Technology (NIST), and the International Atomic Energy Agency (IAEA), and all were directly traceable to the primary isotopic reference material for each element (i.e., VPDB for δ^13^C and VSMOW for δ^2^H). Calibration procedures for CSIA of FAMEs were applied identically across reference and sample materials. First, the provisional isotopic value for each FAME was obtained by normalization to an isotopically calibrated internal reference compound (e.g., c13:0). Isotopic values of the individual FAMEs were then scale-normalized to the primary reference materials by using FMIX1, an external mixture composed of FAMEs with a broad range of calibrated δ^13^C and δ^2^H values. Final quality assessment was based on the accuracy and precision of an unbiased quality control material, a second δ^13^C- and δ^2^H-calibrated FAMEs mixture, FMIX2. The mean measurement error of sample replicates was ±0.16 and ±2.3 for δ^13^C and δ^2^H, respectively, while the measurement accuracy, as determined by quality control materials, was within ±0.43 and ±3.1 for δ^13^C- and δ^2^H, respectively.

### CSIA—δ^13^C, δ^15^N and δ^2^H of Amino Acids

The experimental procedure followed several previous works.^32−34^ Peeled walnut kernels were hydrolyzed (6 M HCl for 70 min at 150 °C under a N_2_ headspace). The amino acids were later derivatized as *N*-acetyl methyl esters (NACME). NACME was analyzed via GC-combustion-IRMS (GC-C-IRMS, Thermo Trace GC 1310 gas chromatography coupled to a Thermo Scientific Delta V Advantage isotope ratio mass spectrometer via a GC IsoLink II Combustion Interface).

Sample injection was done at 260 °C in splitless mode for 1 min. The separation was performed on an Agilent DB-35 column (60 m × 0.32 mm ID × 1.5 μm film thickness) with a constant flow rate of 2 mL/min. The column temperature was set as follows: 70 °C (hold 2 min); 140 °C (15 °C/min, hold 4 min); 240 °C (12 °C/min, hold 5 min); and 255 °C (8 °C/min, hold 35 min).

The combustion reactor was a NiO tube containing CuO and NiO wires maintained at 1000 °C. Water was removed through a Nafion dryer before the analyte gases were transferred to the IRMS. During ^15^N analysis, CO_2_ was removed from the postcombustion carrier stream using a liquid nitrogen trap to prevent isobaric interferences within the ion source. For ^2^H analysis, individual AAs were converted to H_2_ within a high-temperature thermal conversion reactor of a graphitized Al_2_O_3_ tube maintained at 1425 °C.

All samples were analyzed in duplicate; further replicates were analyzed if the initial measurements fell outside the expected measurement error. Replicates of the quality control and assurance reference materials were measured for every five samples.

The pure amino acids used in quality control and assurance mixtures had been calibrated separately by EA-IRMS and were directly traceable to the primary isotopic reference material for each element (i.e., VPDB for δ^13^C, Air for δ^15^N, and VSMOW for δ^2^H). EA/HTC-IRMS was performed using secondary reference materials calibrated against certified standard reference materials from USGS, NIST, and the IAEA (i.e., IAEA-600, USGS40, USGS41, USGS42, USGS43, USGS61, USGS64, and USGS65). Calibration procedures for CSIA of amino acids were applied identically across reference and sample materials. First, initial isotopic values for all amino acids were adjusted such that the known isotopic composition of an internal reference material (e.g., Nor) was obtained. Next, the isotopic values of the individual amino acids were adjusted based on the first quality assurance reference mixture, UCD AA1, such that the known isotopic composition of each amino acid within the mixture was obtained. Finally, measurements were scale-normalized to the primary reference materials for δ^15^N (i.e., Air) using the second quality assurance reference mixture, UCD AA2; in the case of δ^13^C analysis, calibration then proceeded by accounting for the influence of exogenous carbon and potential kinetic isotope effects following derivatization. For ^2^H analysis, scale normalization of individual AAs was performed using certified *n*-alkanes supplied by Indiana University (IU A7). Final quality assessment was based on the accuracy and precision of unbiased quality control materials, which included a calibrated amino acid mixture, UCD AA3, and multiple natural materials.

The mean measurement error of sample replicates was ±0.26, ±0.61, ±6.7 for δ^13^C, δ^15^N and δ^2^H, respectively, while the measurement accuracy, as determined by quality control materials, was within ±0.56, ±0.53, ±11.8 for δ^13^C, δ^15^N and δ^2^H, respectively.

### Statistical Analysis

The stable carbon isotope values were expressed in δ notation which illustrated the deviations of the isotope ratio of a sample relative to a standard, for instance
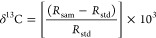
where *R*_sam_ and *R*_std_ are the ^13^C/^12^C of the sample and standard, respectively.

Analysis of variance (ANOVA) was used to determine if means differed significantly between the USA and China. Linear discriminant analysis (LDA) was performed for dimensionality reduction. All of the analysis was performed using R Studio 2022.07.01 (Posit software, PBC, Boston, MA) and R 4.2.1 (The R Foundation for Statistical Computing, Vienna, Austria).

## Results and Discussion

### BSIA—δ^13^C, δ^15^N, and δ^34^S

BSIA of walnut kernels from multiple countries had ranges of −29.30 to −24.81‰ and −2.00 to 7.34‰ for δ^13^C_bulk_ and δ^15^N_bulk_, respectively (Table 1). These ranges were similar to those reported on samples from Germany and France.^27^ δ^34^S_bulk_ was −0.64 to 16.70‰ (Table 1). No data have been reported on δ^34^S of walnuts in the literature.

**Table 1:** δ^13^C_bulk_, δ^15^N_bulk_, and δ^34^S_bulk_ (‰) of Walnut Kernels from China (Ch), Moldova (Mo), Poland (Po), the USA (US), India (In), Romania (Ro), and Ukraine (Uk)^a^

	δ^13^C, δ^15^N, and δ^34^S (‰)								
element	US		Ch		Mo	Po	In	Ro	Uk
S	9.46 ± 4.34	a	5.54 ± 3.95	a	5.52	5.54	3.36	3.54	5.85
C	–27.23 ± 1.00	a	–26.67 ± 1.39	a	–25.56	–27.35	–26.14	–27.08	–24.81
N	1.29 ± 1.59	a	1.28 ± 1.50	a	6.60	4.67	6.90	6.41	6.95

a Different letters for the same parameter indicating significant difference among the countries (*p* ≤ 0.05).

Our data suggested that δ^13^C_bulk_ and δ^34^S_bulk_ were not significantly different (*p* > 0.05) between the samples from the USA and China (Table 1). δ^13^C_bulk_ is mainly used to distinguish photosynthetic pathways (C_3_, C_4_, or CAM),^15^ with C_3_ plants having significantly lower values than C_4_ plants. C3 plants use Calvin cycle, C4 plants employ Hatch–Slack cycle, while CAM plants can utilize both depending on the conditions.^13^ All of the samples in this study belong to the same species (*Juglans regia* L.) and are C_3_ plants;^27^ therefore, the lack of intraspecific variation in δ^13^C_bulk_ values is not surprising. While there have been studies indicating that δ^13^C_bulk_ may respond to geographical features like altitude, climatic moisture, and temperature of the growing locations in whitebark pines^35^ and wheat,^36^ it was not noticeable in this study and some others.^37^

While Mahalovich et al.^26^ found a correlation between δ^34^S_bulk_ values and climate (summer mean annual temperature, frost-free period, and annual precipitation),^35^ the δ^34^S_bulk_ values of walnuts were not significantly different among the countries, whereas variation within countries (i.e., within China or USA) was high. The intense within-country variation has also been observed in tomato and its paste.^11^ The sources of δ^34^S_bulk_ variability can stem from fertilizer types and quantity,^10^ with further modification via enzymatic sulfur isotope fractionation.^30^

Like δ^13^C_bulk_ and δ^34^S_bulk_, δ^15^N_bulk_ values of China and the USA samples were not significantly different (*p* > 0.05). However, they were lower than those from other countries (Table 1). Many previous studies have demonstrated that reduced δ^15^N_bulk_ values are associated with inorganic fertilizers common to conventional agriculture, whereas higher δ^15^N_bulk_ results from the use of organic sources of N, such as manures, in organic farming.^10^ This finding is expected, as the majority of walnuts grown in the USA are produced through conventional practice^38^ and many walnut orchards in China also rely on synthetic fertilizer.^39^ The lower δ^15^N_bulk_ values associated with synthetic fertilizer corresponded to minimum or no isotope fractionation occurring during the production of the fertilizer via the Haber–Bosch process.^13^

High standard deviations within the Chinese and the USA samples (Table 1) indicated the variation in the level of synthetic fertilizer usage and availability of other primary nitrogen sources besides synthetic fertilizer, i.e., soil nitrogen.^38^ δ^13^C and δ^15^N could also be influenced by cadmium exposure to plants, e.g., in castor,^39^ but it was not evident in our study.

These findings reflected that the BSIA data of δ^13^C, δ^15^N, and δ^34^S were not sufficient for reliable discrimination of the walnut samples based on the geographical provenance. This could result from the fluctuation of the values among different chemical groups present in the samples that eventually compensated the BSIA values. Therefore, CSIA should be employed as it has been proven to be more sensitive in responding to geographical changes. Another point to consider is analyzing BSIA of different portions of walnut kernels or walnut plants. For example, a study on peanuts showed that utilizing defatted portions improved the classification.^40^ This way, the risk of values becoming evened out, e.g., by the values of lipid compounds, is less than in the whole kernel, although still not as specific as CSIA.

#### CSIA—Fatty Acids

##### CSIA—δ^2^H of Fatty Acids

Lipids are the dominant nutrient in walnuts, with concentrations reaching 75/100 g of dry matter. Fatty acids are key components of glycoglycerolipids and glycerophospholipids, the major components of walnut lipids.^41^ Stable isotope analysis of fatty acids has been used in other food products to successfully determine geographical provenance^42^ and differentiate organic from conventional produce.^43^ This is possible due to the environmental effects on isotope fractionation during lipid metabolism.

Isotope fractionation of hydrogen in lipids is reflected in biochemical, physiological, and environmental influences. To the best of our knowledge, this is the first study analyzing δ^2^H_fatty acids_ in walnuts from multiple regions. A sample chromatogram of ^2^H_fatty acid_ is given in Figure S2a in the Supporting Information (SI). The range of δ^2^H_fatty acids_ (−249.89 to −80.01‰) (Table 2) was wider than the range for δ^2^H_bulk_ of walnuts grown in Germany and France (−186 to −147‰).^27^ This is unsurprising, as δ^2^H_bulk_ reflects the sum of CSIA values of all compounds, and the variation among them were high and could compensate the BSIA values. Wider variation in isotope values of constituent molecules compared to the bulk tissue is tied to their specific biochemistries and is one of the reasons why CSIA is more well suited for discrimination purposes.

**Table 2:** δ^2^H (‰) of Fatty Acids in Walnuts Sourced from Seven Countries: China (Ch), Moldova (Mo), Poland (Po), the USA (US), India (In), Romania (Ro), and Ukraine (Uk)^a^^,^^b^

	δ^2^H (‰) of fatty acids										
fatty acid	US			Ch			Mo	Po	In	Ro	Uk
C16:0	–189.22 ± 9.59	A	b	–189.87 ± 11.48	A	b	–200.48	–212.09	–170.62	–199.77	–202.75
C18:1w9c	–109.08 ± 12.30	A	a	–131.02 ± 16.42	B	a	–143.06	–162.44	–118.68	–154.43	–173.96
C18:2w6c	–218.76 ± 9.31	A	c	–221.72 ± 12.76	A	c	–227.84	–242.44	–202.32	–225.15	–238.18
C18:3w3c	–228.46 ± 15.38	A	d	–230.13 ± 10.31	A	c	–247.59	–232.19	–218.96	–250.63	–257.98

a Different capital letters for the same parameter indicating significant difference among the countries (*p* ≤ 0.05).

b Different small letters for the same country indicating significant difference among the parameters (*p* ≤ 0.05).

Significant difference (*p* < 0.05) between the USA and China samples was only observed on δ^2^H_C18:1*n*–9_. For δ^2^H_C16:0_, δ^2^H_C18:2*n*–6_, and δ^2^H_C18:3*n*–3_, the sample from India had the highest δ^2^H_fatty acids_ value, followed by the samples from the USA and China. Interestingly, δ^2^H_C18:1*n*–9_ showed a distinct profile: the USA samples had the highest average value than the others, followed by the Indian and Chinese samples (Table 2).

δ^2^H values are influenced by several geographical factors, including precipitation/rainfall level, temperature, elevation, distance from coast,^44^ and whether surface water and/or groundwater is used in cultivation.^45^ Using surface water (e.g., irrigation) led to increased δ^2^H values, whereas more negative values were associated with groundwater use.^45^ Many walnut orchards in the USA and China are irrigated;^46^ therefore, the higher values of δ^2^H of the USA and Chinese samples, as compared to those of Moldova, Poland, Romania, and Ukraine, are logical. The average values for the USA samples were slightly larger than those of China, probably due to more intense irrigation.

For δ^2^H_C16:0_, δ^2^H_C18:2*n*–6_, and δ^2^H_C18:3*n*–3_, the value of the India sample exceeded those of the USA. We did not have any information about whether it was irrigated during its cultivation. The higher value could not be explained by the continental effect because the northwestern Himalayan regions, particularly Jammu and Kashmir, are the main areas of walnut cultivation in India.^47^ These areas are at a farther distance from the nearest coast (±1200 km), compared to the Central Valley-CA, where all of the USA samples originated, to the nearest coast (±140 km). Precipitation that occurs at a farther distance from a coast is generally more depleted in δ^2^H than the precipitation near a coast. Altitude effects may be more reasonable in explaining this finding. A previous study reported a positive correlation between altitude and δ^2^H.^48^ This theory supported our data because the approximate altitude/elevation of the Central Valley and northwestern Himalaya are 50 and 1200–3500 m, respectively.

The fact that oleic acid showed distinct characteristics for country comparison, at which the USA samples were more enriched than the India sample and those of the other countries, could be related to the more intense desaturation process of oleic acid to generate more polyunsaturated fatty acids. This was evidenced by the lower ratio of oleic acid (the main monounsaturated fatty acid) to polyunsaturated fatty acids of the USA samples, as compared to those of other countries (Figure S1, SI).

Oleic acid was more enriched in δ^2^H than linoleic acid and linoleic acid was more enriched than linolenic acid for most of the samples (Table 2). This is not unexpected, as numerous studies have found biochemical isotope fractionation results in substrates that are more enriched than their products.^14^ Oleic acid acts as a substrate for linoleic acid synthesis, whereas linoleic acid is the substrate for linolenic acid. Serial isotopic depletion of oleic acid desaturation products has also been observed elsewhere.^41^

##### CSIA—δ^13^C of Fatty Acids

We analyzed δ^13^C_fatty acid_ and compared δ^13^C_fatty acid_ in samples sourced from multiple countries. A sample chromatogram of ^13^C_fatty acid_ is provided in Figure S2b, SI. The predominant fatty acids of walnuts were C16:0, C18:0, C18:1*n* – 9, C18:2*n* – 6, and C18:3*n* – 3, and they had a range of carbon stable isotope values typical of C_3_ plants (Table 3). The values for δ^13^C_C16:0_, δ^13^C_C18:0_, and δ^13^C_C18:1*n*–9_ were within the range of δ^13^C previously reported on walnut oils from China. The δ^13^C_C18:2*n*–6_ values were also mostly within the reported range with a few exceptions. For δ^13^C_C18:3*n*–3_, many of our data were slightly higher than those reported in that study.^28^

**Table 3:** δ^13^C (‰) of Fatty Acids in Walnuts Sourced from Seven Countries: China (Ch), Moldova (Mo), Poland (Po), the USA (US), India (In), Romania (Ro), and Ukraine (Uk)^a^^,^^b^

	δ^13^C (‰) of fatty acids										
fatty acid	US			Ch			Mo	Po	In	Ro	Uk
C16:0	–30.51 ± 0.69	A	b	–30.15 ± 0.56	A	b	–30.19	–30.94	–30.13	–30.21	–29.96
C18:0	–31.20 ± 0.75	A	c	–30.56 ± 0.56	A	b	–30.35	–30.22	–30.21	–30.44	–30.58
C18:1w9c	–29.93 ± 0.73	A	a	–29.38 ± 0.78	A	ab	–29.07	–29.86	–29.18	–29.13	–29.38
C18:2w6c	–29.68 ± 0.70	B	a	–28.42 ± 0.72	A	a	–28.67	–29.33	–28.39	–28.30	–28.18
C18:3w3c	–30.88 ± 0.79	B	bc	–29.8 ± 0.85	A	b	–29.89	–30.95	–30.32	–29.62	–29.72

a Different capital letters for the same parameter indicate significant difference among the countries (*p* ≤ 0.05).

b Different small letters for the same country indicate significant difference among the parameters (*p* ≤ 0.05).

Minor variation was observed among fatty acids from the same country of origin. For the USA samples, δ^13^C_C18:2*n*–6_ had the highest level, followed by δ^13^C_C18:1*n*–9_, δ^13^C_C16:0_, δ^13^C_C18:3*n*–3_, and then δ^13^C_C18:0_. A similar pattern was noticed in Chinese samples, except that δ^13^C_C18:3*n*–3_ had a slightly higher value, albeit insignificant, than δ^13^C_C16:0_ (Table 3). A previous study of olive oils showed that in oils from unripe olives, the carbon stable isotope ratios of oleic and linoleic acids were significantly different, whereas no noticeable difference was found in oils from ripe fruits.^49^ It is likely that linoleic and linolenic acids start to break down into volatiles in ripe walnuts during advanced ripening and lead to a smaller proportion of polyunsaturated fatty acids.^50^

Due to isotope fractionation, elongation from palmitic to stearic acid yielded a slight δ^13^C depletion of the later compound in most of the samples (Table 3). However, δ^13^C_fatty acids_ of the desaturation products of stearic acid were confounded because the products were slightly more enriched (δ^13^C_C18:2*n*–6_ > δ^13^C_C18:1*n*–9_ > δ^13^C_C18:0_). Similar trends have been observed in pumpkin oil^51^ and various seed oils^52^ because unsaturated fatty acids are prone to oxidation.

Our data showed that there was significant difference in δ^13^C_C18:2*n*–6_ and δ^13^C_C18:3*n*–3_ between the USA and China samples but not for the other fatty acids (Table 3). This confirmed earlier findings that cultivation locations had some effects on the carbon stable isotopes of individual fatty acids.^28^ The earlier study on walnut oils showed a significant difference in δ^13^C values of all dominant fatty acids among samples from different regions in China. Noticeable difference in δ^13^C_C18:2*n*–6_ and δ^13^C_C18:3*n*–3_ values between China and the USA samples may originate from varying intensity of the fatty acid oxidation because they are both polyunsaturated fatty acids. Relatively higher values in the China samples could indicate that they were oxidized at a faster rate.

These two fatty acids were likely more sensitive to environmental factors affecting δ^13^C values, e.g., temperature and water availability. These factors can influence stomatal aperture and CO_2_ diffusion into leaves.^13^ The higher sensitivity of these fatty acids was reasonable, considering that their synthesis included more reactions than the others.

#### CSIA—Amino Acids

##### CSIA—δ^13^C of Amino Acids

CSIA of amino acids had previously been used in other matrices to evaluate resource utilization and trophic connections among organisms in ecosystems^53^ and authenticity of organic produce.^11^ This is the first study reporting CSIA-amino acids in walnuts. A sample chromatogram of ^13^C_amino acid_ is provided in Figure S2c, SI. Most δ^13^C_amino acid_ values were higher than δ^13^C_fatty acids_, agreeing with previous studies showing that δ^13^C_carbohydrate_ > δ^13^C_protein_ > δ^13^C_lignin_ > δ^13^C_lipid_.^13^

For walnut kernels, δ^13^C_His_, δ^13^C_Hyp_, and δ^13^C_Met_ were present at concentrations lower than the limit of quantitation for all of the samples and are not included here. Among all of the walnut amino acids, δ^13^C_Ser_ had the highest value. In general, δ^13^C_amino acid_ values were moderately higher than δ^13^C_bulk_, except for leucine, phenylalanine, tyrosine, and valine. δ^13^C_Phe_ and δ^13^C_Val_ were more depleted, while δ^13^C_Leu_ and δ^13^C_Tyr_ were like the δ^13^C_bulk_ (Tables 1 and 4). The difference between δ^13^C_amino acid_ and δ^13^C_bulk_ is not surprising, as bulk was not lipid-extracted.

**Table 4:** δ^13^C (‰) of Amino Acids in Walnuts Sourced from Seven Countries: China (Ch), Moldova (Mo), Poland (Po), the USA (US), India (In), Romania (Ro), and Ukraine (Uk)^a^^,^^b^

	δ^13^C (‰) of amino acids										
amino acid	US			Ch			Mo	Po	In	Ro	Uk
Ala	–20.32 ± 1.31	A	e	–19.99 ± 1.67	A	cd	–19.85	–20.35	–19.04	–20.65	–19.5
Asx	–18.73 ± 1.16	A	d	–18.18 ± 1.04	A	bc	–18.18	–18.31	–18.41	–18.87	–17.4
Glx	–20.55 ± 1.02	A	e	–20.64 ± 1.04	A	cd	–20.25	–21.41	–20.55	–21.16	–20.11
Gly	–20.74 ± 1.71	B	e	–19.47 ± 1.64	A	cd	–19.69	–21.04	–19.85	–20.10	–19.32
Ile	–22.79 ± 1.22	A	f	–21.75 ± 0.99	A	d	–21.75	–22.37	–21.28	–22.75	–21.31
Leu	–28.10 ± 1.12	B	g	–26.55 ± 1.04	A	e	–26.58	–27.58	–27.76	–27.75	–25.98
Lys	–16.05 ± 2.88	A	c	–17.91 ± 0.97	A	bc	–18.36	–18.15	–18.36	–19.1	–17.66
Phe	–31.11 ± 1.21	A	h	–30.83 ± 1.52	A	g	–30.45	–31.15	–29.01	–31.64	–28.64
Pro	–19.74 ± 1.20	A	de	–20.03 ± 1.36	A	cd	–19.92	–20.66	–19.84	–20.45	–19.22
Ser	–12.50 ± 1.61	A	a	–13.35 ± 1.26	A	a	–12.52	–13.27	–12.56	–13.28	–11.31
Thr	–13.95 ± 1.77	A	b	–15.43 ± 1.66	A	ab	–14.96	–16.16	–14.17	–16.25	–14.68
Tyr	–27.62 ± 1.93	A	g	–27.22 ± 1.11	A	ef	–26.71	–27.63	–27.16	–27.93	–25.96
Val	–30.56 ± 1.25	B	h	–29.45 ± 1.07	A	fg	–29.60	–30.23	–30.6	–30.67	–29.37

a Different capital letters for the same parameter indicate significant difference among the countries (*p* ≤ 0.05).

b Different small letters for the same country indicate significant difference among the parameters (*p* ≤ 0.05).

Because there are no previous studies on walnuts on this parameter, we compare the data to those of other food products. The range of our δ^13^C_amino acid_ values overlapped with that reported for rice in South Korea^43^ and tomatoes in Italy.^11^ While some values were similar, the other δ^13^C_amino acid_ values of walnuts were higher than the values for tomatoes and rice. This was reasonable because of the genetic variation.

ANOVA results showed that no significant difference (*p* > 0.05) was observed among the means of δ^13^C_amino acid_ between the USA and China samples, except for glycine, leucine, and valine (Table 4). Interestingly, these three compounds are grouped into the same category: aliphatic amino acid. Similar findings were observed in a shiitake authentication study. δ^13^C_leucine_ discriminated shiitake mushrooms that were grown organically to those grown conventionally, at which the values were more negative. Meanwhile, δ^13^C_valine_ of organic shiitake was significantly lower than the pesticide-free and conventional shiitake.^25^ A study on rice demonstrated no significant difference in δ^13^C_leucine_ and δ^13^C_valine_ among organic, pesticide-free, and conventional rice but the values for δ^13^C_isoleucine_, δ^13^C_lysine_, and δ^13^C_tyrosine_ were significantly different. The organic sample had more negative values than the conventional ones, except for δ^13^C_tyrosine_ that showed a reverse effect. A study on tomatoes found a significant difference in δ^13^C_Glx_ when organic and conventional tomatoes were compared, but no differences in the remaining δ^13^C_amino acid_.

The tomato study described that samples grown in two locations had similar δ^13^C_amino acids_. δ^13^C_amino acids_ of tomatoes grown in different years were also not significantly different.^11^ Taken together, this may indicate that location and cultivation time (and possible climate variability associated with it) had a minimum effect on δ^13^C_amino acids_. However, in our study, there were small but significant differences in the δ^13^C_amino acids_ profile between samples from China and the USA. It showed that this parameter had some utility for origin discrimination, similar to δ^13^C_fatty acid_, but the reasons need to be explored more. It could be related to geographical factors affecting the stomatal aperture and subsequent photosynthesis.

##### CSIA—δ^15^N of Amino Acids

Nitrogen stable isotope analysis of amino acids had been studied in other food matrices like rice^43^ and tomatoes,^11^ mainly to distinguish organic vs conventional or pesticide-free products. A sample chromatogram of ^15^N_amino acid_ is displayed in Figure S2d, SI.

Similar to the carbon amino acids, δ^15^N_His_, δ^15^N_Hyp_, and δ^15^N_Met_ were present in amounts below the limit of quantification in all of the samples and are not included here. Some δ^15^N_amino acids_, for instance, δ^15^N_Phe_, δ^15^N_Leu_, were lower than δ^15^N_bulk_ while others (e.g., δ^15^N_Asx_, δ^15^N_Lys_, and δ^15^N_Pro_) were higher than δ^15^N_bulk_ (Table 5), similar to a study in shiitake mushroom.^25^ The difference among δ^15^N_amino acid_ reflected well-known differences in nitrogen isotope fractionation during nitrogen assimilation as well as synthesis and metabolism of amino acids. Enrichment, relative to the δ^15^N_bulk_ values, might indicate that the amino acids further converted into other compounds, e.g., catabolism of lysine in response to biotic and abiotic stress or increased concentration of this compound. Proline accumulation occurred when plants were exposed to stress. Depletion, relative to the bulk, might signal the lengthy process during their synthesis.^54^

**Table 5:** δ^15^N (‰) of Amino Acids in Walnuts Sourced from Seven Countries: China (Ch), Moldova (Mo), Poland (Po), the USA (US), India (In), Romania (Ro), and Ukraine (Uk)^a^^,^^b^

	δ^15^N (‰) of amino acids										
amino acid	US			Ch			Mo	Po	In	Ro	Uk
Ala	0.86 ± 1.58	A	cdef	1.78 ± 1.86	A	a	5.85	3.72	3.96	6.15	6.08
Asx	2.2 ± 1.49	A	abc	2.75 ± 2.03	A	a	7.27	5.74	6.34	7.72	7.98
Glx	1.74 ± 1.48	A	bcde	2.06 ± 2.08	A	a	6.73	5.11	6.33	7.2	7.31
Gly	1.43 ± 1.77	A	cde	2.53 ± 2.88	A	a	7.46	3.89	5.39	7.41	8.09
Ile	0.38 ± 1.87	A	ef	0.41 ± 2.34	A	a	5.42	3.01	3.71	5.2	5.6
Leu	–0.57 ± 1.47	A	fg	–0.57 ± 2.23	A	a	4.21	2.05	3.19	4.35	4.32
Lys	1.96 ± 1.78	A	abcd	2.93 ± 3.24	A	a	7.73	6.06	8.05	8.38	7.75
Phe	–1.31 ± 1.97	A	g	–0.55 ± 2.37	A	a	3.51	2.05	1.41	3.68	4.89
Pro	3.41 ± 1.57	A	a	3.9 ± 2.37	A	a	8.65	7.30	7.45	8.6	8.78
Ser	1.38 ± 1.66	A	cde	1.86 ± 2.97	A	a	6.21	2.97	4.15	5.77	6.15
Thr	0.56 ± 1.92	A	def	1.22 ± 3.67	A	a	6.02	7.06	5.03	6.64	5.52
Tyr	1.04 ± 1.85	A	cde	2.42 ± 2.60	A	a	6.06	5.79	5.11	7	8.57
Val	2.93 ± 1.55	A	ab	3.32 ± 2.60	A	a	8.72	6.92	8.27	8.89	9.11

a Different capital letters for the same parameter indicate significant difference among the countries (*p* ≤ 0.05).

b Different small letters for the same country indicate significant difference among the parameters (*p* ≤ 0.05).

In general, the USA samples had the lowest values for all of the amino acids while Moldova had the highest values. For all of the amino acids investigated, Chinese samples were not significantly different from the USA samples (*p* > 0.05). This follows the δ^15^N_bulk_ data, indicating that most walnuts from these two countries were grown mainly using a similar method in which synthetic fertilizer was used. This is supported by a prior finding in tomato and shiitake where δ^15^N_amino acid_ values were lower in tomatoes grown using the conventional method.^25^ Synthetic fertilizer is commonly produced via the Haber–Bosch reaction that relies on nitrogen fixation from the atmosphere; therefore, the δ^15^N values are close to 0‰. Green manure from legumes also exhibits nitrogen values near atmophere; however, animal manure and compost undergo more complex biochemical reactions resulting in higher δ^15^N.^55^ Thus, we hypothesized that the samples from other countries besides China and the USA used compost or animal manure.

Across all amino acids, valine had the highest δ^15^N values, except for proline in some cases (Table 5). In contrast, phenylalanine and leucine had the lowest δ^15^N values among the walnut δ^15^N_amino acid_. This is similar to previous findings from tomato, komatsuna, and spinach.^11,56^ The synthesis of these amino acids is a complex process involving the shikimate pathway and other amino acids.^57^

Positive values of Δ_amino acid Glx_ (δ^15^N_amino acid_ – δ^15^N_Glx_) were found for aspartate, lysine, proline, and valine (for the USA samples) and aspartate, glycine, lysine, proline, tyrosine, and valine (for the China samples). Negative fractionation occurred during the synthesis of alanine, glycine, isoleucine, leucine, phenylalanine, serine, threonine, and tyrosine (for the USA samples) and alanine, isoleucine, leucine, phenylalanine, serine, and threonine (for the China samples). This profile shared some similarity with rice^43^ and shiitake,^25^ but some contrasts were also noticed that supported the idea that isotope fractionation of amino acids is influenced by genetics.^25^

Similar to carbon of the same compound group, the nitrogen stable isotope of amino acids is rarely used to distinguish geographical origins. They are mainly used to investigate agricultural practices, particularly, types of fertilizer use. In this study, δ^15^N_amino acids_ showed very strong correlation with δ^15^N_bulk_, indicating that δ^15^N_bulk_ data was sufficient if investigation of fertilizer type (i.e., N source) was the sole purpose of the study.

##### CSIA—δ^2^H of Amino Acids

δ^2^H_amino acids_ were evaluated on the samples from China and the USA but not from those of the other countries. A sample chromatogram can be found in Figure S2e, SI. There were four amino acids with a significant difference among the USA and China samples: glutamate, isoleucine, leucine, and threonine (Table 6). For the first three compounds, the values in the China samples were lower, but for threonine, the opposite trend was observed. Lower δ^2^H_amino acids_ values generally corresponded to a higher distance from the nearest coast (continental effect) as precipitation that occurs at a farther area from the sea is more depleted in ^2^H.^13^ Central Valley, the main walnut growing area in the USA, is around 140 km from the nearest coast, whereas Yunnan, Shaanxi, and Xinjiang, the sources of our China samples, are at approximately 900, 1100, and 3000 km from the nearest coast, respectively. The reverse trend on threonine could be confounded by its conversion into other amino acids.

**Table 6:** δ^2^H (‰) of Amino Acids in Walnuts Sourced from China (Ch) and the USA (US)^a^

	δ^2^H (‰) of amino acids			
amino acid	US		Ch	
Ala	–106.29 ± 4.63	A	–106.89 ± 13.08	A
Asx	–106.03 ± 3.87	A	–109.94 ± 9.36	A
Glx	–96.71 ± 4.56	A	–103.89 ± 13.15	B
Gly	–111.87 ± 4.06	A	–116.01 ± 8.94	A
Ile	–246.08 ± 7.10	A	–258.50 ± 11.69	B
Leu	–170.08 ± 5.57	A	–179.73 ± 14.71	B
Lys	–155.55 ± 9.85	A	–157.80 ± 9.74	A
Met	–127.18 ± 18.57	A	–132.17 ± 9.98	A
Phe	–93.58 ± 7.60	A	–95.07 ± 11.38	A
Pro	–84.86 ± 7.14	A	–92.69 ± 14.27	A
Ser	–78.80 ± 5.32	A	–82.53 ± 10.64	A
Thr	–192.09 ± 4.03	B	–186.80 ± 6.97	A
Tyr	–104.04 ± 5.81	A	–98.65 ± 9.17	A
Val	–190.69 ± 7.45	A	–197.05 ± 14.38	A

a Different capital letters for the same parameter indicate significant difference among the countries (*p* ≤ 0.05).

Our data demonstrates that δ^2^H_amino acids_ were generally more enriched than δ^2^H_fatty acids_, agreeing with a previous study on avocado.^58^ A plausible reason was that less isotope fractionation occurred during amino acid synthesis or a more intense catabolism of the group.

### Combination of Multiple Parameters

Our finding demonstrated that BSIA data have limited use in geographical provenance. The values of δ^15^N_bulk_ of the USA and China samples were very different from those of other countries, consistent with modern agricultural practice, i.e., synthetic fertilizer usage. It was not useful, however, to distinguish samples from China to the USA.

CSIA, of both amino acids and fatty acids, showed promising results with a significant difference between samples of the two countries for the following parameters: δ^2^H_C18:1*n*–9_, δ^13^C_C18:2*n*–6_, δ^13^C_C18:3*n*–3_, δ^13^C_Gly_, δ^13^C_Leu_, δ^13^C_Val_, δ^2^H_Glu_, δ^2^H_Ile_, δ^2^H_Leu_, and δ^2^H_Thr_. Similar to the δ^15^N_bulk_, δ^15^N_amino acid_ profiles of the samples from the USA and China were identical, likely due to the same fertilizer type.

**Figure 1:**
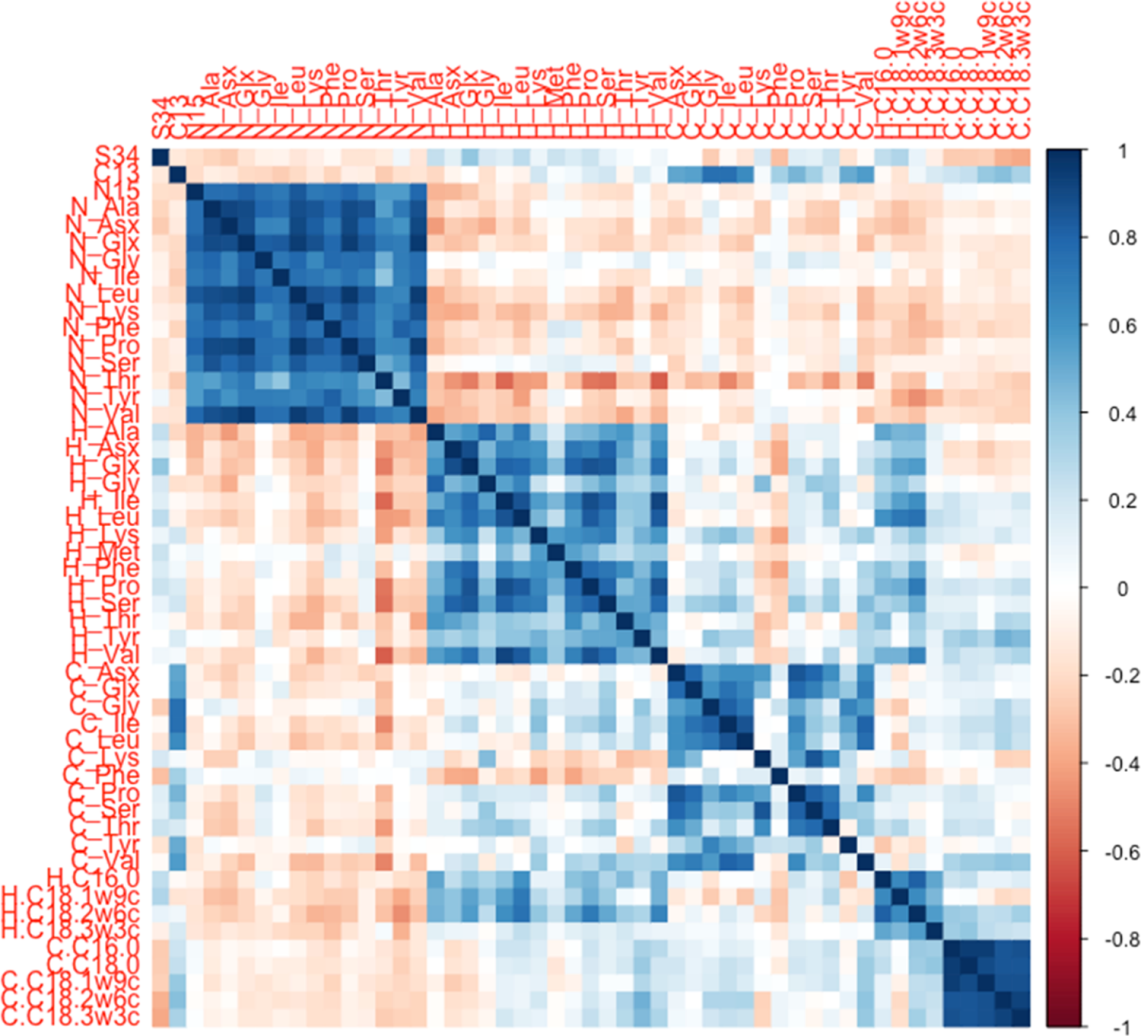
Correlation plot for all of the parameters studied with a significance level of 0.05.

A Pearson correlation test showed that strong positive correlation was observed between δ^15^N_bulk_ and δ^15^N_amino acids_; δ^13^C_bulk_ and δ^13^C_Gly_; δ^13^C_bulk_ and δ^13^C_Ile_; δ^2^H_Leu_ and δ^2^H_C18:2*n*–6_; and ^2^H_Leu_ and δ^2^H_C18:3*n*–3_. High positive correlation was also observed among the majority of δ^15^N_amino acids_ and δ^13^C_fatty acids_ and some of δ^2^H_amino acid_; δ^13^C_amino acid_; and δ^2^H_fatty acid_ values (Figure 1). High correlation between δ^15^N_bulk_ and δ^15^N_amino acids_ and among δ^15^N_amino acids_ was expected considering that synthesis of all amino acids involves either nitrate as the substrate or other amino acid.^59^

Linear discriminant analysis (LDA) was used to evaluate if the stable isotope profiles of the USA and China samples are distinguishable. First, we conducted the test on the CSIA-fatty acid data and found that the profiles of the two countries were different, indicated as separate peaks (no overlapping). There were at least two peaks representing the profile of the China samples, suggesting high variability. The high variability was common for areas as diverse as China (e.g., desert areas in Xinjiang and humid regions of Yunnan). The variations, however, did not compensate for the contrast to the USA samples. When LDA was conducted on either δ^2^H_fatty acids_ or δ^13^C_fatty acids_, overlapping was observed, indicating their insufficiency for the geographical provenance purpose when used separately.

**Figure 2:**
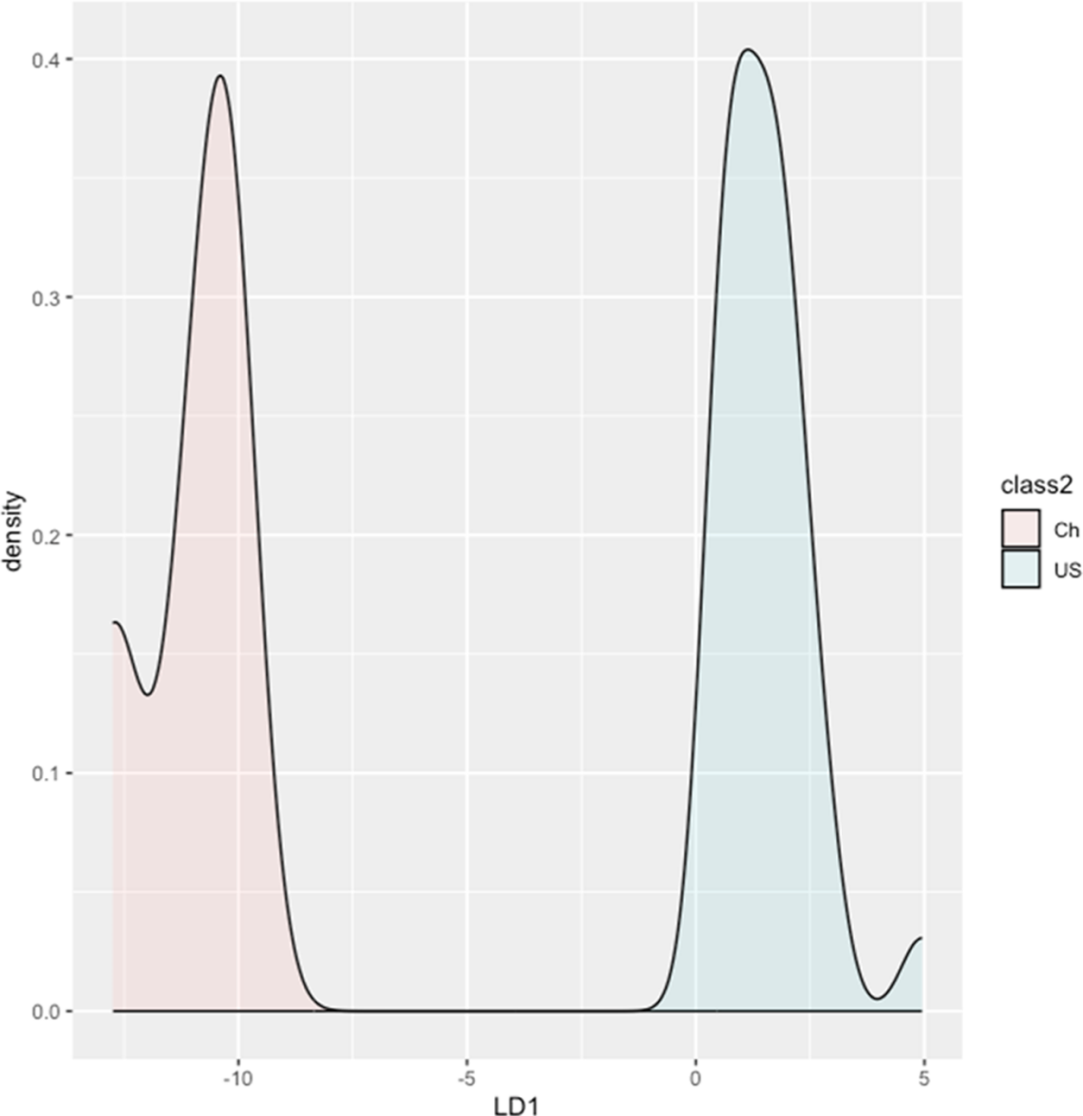
Density plot of the δ^13^C_amino acids_, δ^15^N_amino acids_, and δ^2^H_amino acids_ of samples from China and the USA.

δ^2^H_amino acids_, on the other hand, was enough to separate the samples from the two countries. Identical to the LDA density plot for δ^2^H_fatty acids_ combined with δ^13^C_fatty acids_, there were at least two peaks for the China samples. When δ^13^C, δ^15^N, and δ^2^H_amino acids_ were combined, the discrimination power was greater, expressed as a greater distance between the peaks. When all CSIA values were used together, the LDA density plot showed a clear distinction, but the distances of the USA and China peaks were smaller than that of the combination of δ^13^C_amino acids_, δ^15^N_amino acids_, and δ^2^H_amino acids_. In addition, we also performed LDA on parameters with significant variation only (δ^2^H_C18:1*n*–9_, δ^13^C_C18:2*n*–6_, δ^13^C_C18:3*n*–3_, δ^13^C_Gly_, δ^13^C_Leu_, δ^13^C_Val_, δ^2^H_Glu_, δ^2^H_Ile_, δ^2^H_Leu_, and δ^2^H_Thr_), and a striking contrast was found but at a lesser extent, compared to those of a combination of δ^13^C_amino acids_, δ^15^N_amino acids_, and δ^2^H_amino acids_. Deleting δ^15^N_amino acids_ from the data set, considering that they were not significantly different, did not improve the separation of the two countries. In fact, it slightly decreased the separation distance, reflecting that although not significantly different, δ^15^N_amino acids_ contributed to the LDA. All of the LDA results (shown in density plots) are in Figure S3 (SI), except for the one with the most utility displayed below (Figure 2).

Our findings suggested that CSIA, either fatty acids (δ^2^H_fatty acids_ combined with δ^13^C_fatty acids_) or amino acids (δ^2^H_amino acids_ combined with δ^13^C_amino acids_) had utility in separating samples from China and the USA. This confirmed previous reports that carbon and hydrogen stable isotope fractionation was sensitive to variations in geographical features, i.e., distance to coast, elevation, and humidity. The LDA results demonstrated that CSIA-amino acid yielded a larger contrast between the two countries than CSIA-fatty acid. When time and funding were limited, δ^2^H_amino acids_ combined with δ^13^C_amino acids_ or δ^2^H_amino acids_ alone were likely sufficient for discriminating the samples from the two countries. δ^15^N, however, was not useful, most likely because the two countries applied a similar fertilization strategy.

## Supporting Information Available

Ratio of monounsaturated fatty acid to polyunsaturated fatty acids—typical chromatograms—LDA density plots (PDF)

## Abbreviations

BSIA: bulk stable isotope analysis
CSIA: compound-specific stable isotope analysis
Ala: alanine
Asx: asparagine or aspartic acid
Glx: glutamate or glutamine
His: histidine
Hyp: hydroxyproline
Ile: isoleucine
Leu: leucine
Lys: lysine
Met: methionine
Phe: phenylalanine
Pro: proline
Ser: serine
Thr: threonine
Tyr: tyrosine
Val: valine
