# Supplementary material for: Bulk and Compound-Specific Stable Isotope Analysis for the Authentication of Walnuts (Juglans regia) Origins
Source: J Agric Food Chem. 2023 Nov 2;71(45):16939–49. doi: 10.1021/acs.jafc.3c03770 (PMC10655176; doi:10.1021/acs.jafc.3c03770)
Supplement: Supplementary file 1 — jf3c03770_si_001.pdf [file jf3c03770_si_001.pdf]

Supporting document

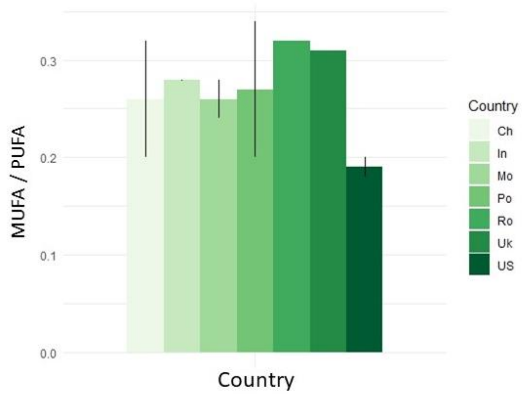

Figure 1. Bar plot of the ratio of monounsaturated fatty acid (particularly oleic acid) to polyunsaturated fatty acids (linoleic and linolenic acids) of samples sourced from China (Ch), India (In), Moldova (Mo), Poland (Po), Romania (Ro), Ukraine (Uk), and the USA (US)

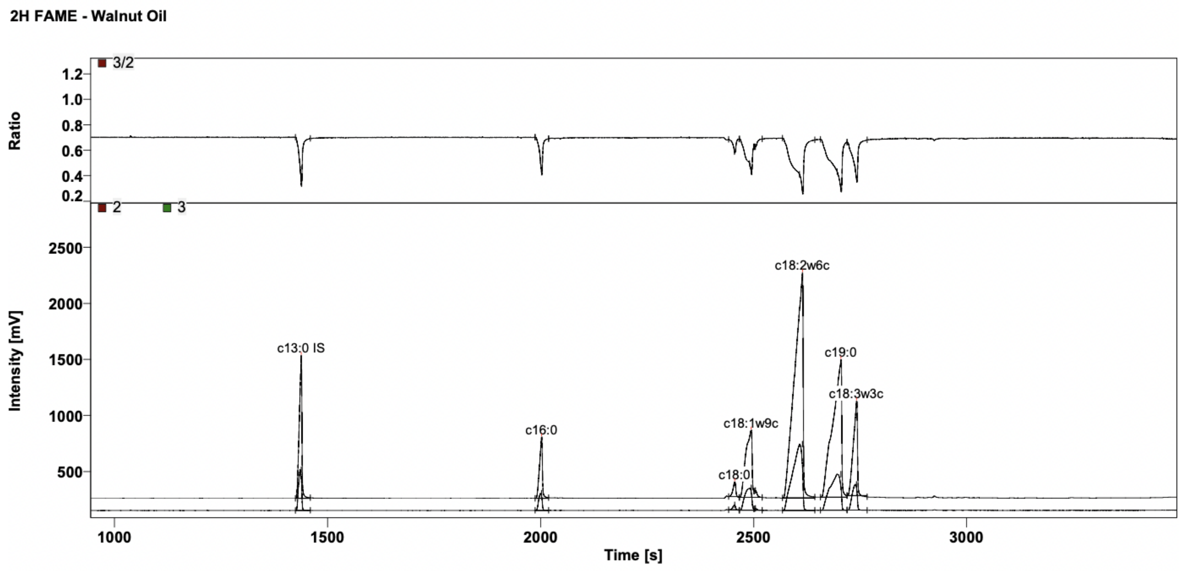

a) ^{2}H of fatty acids

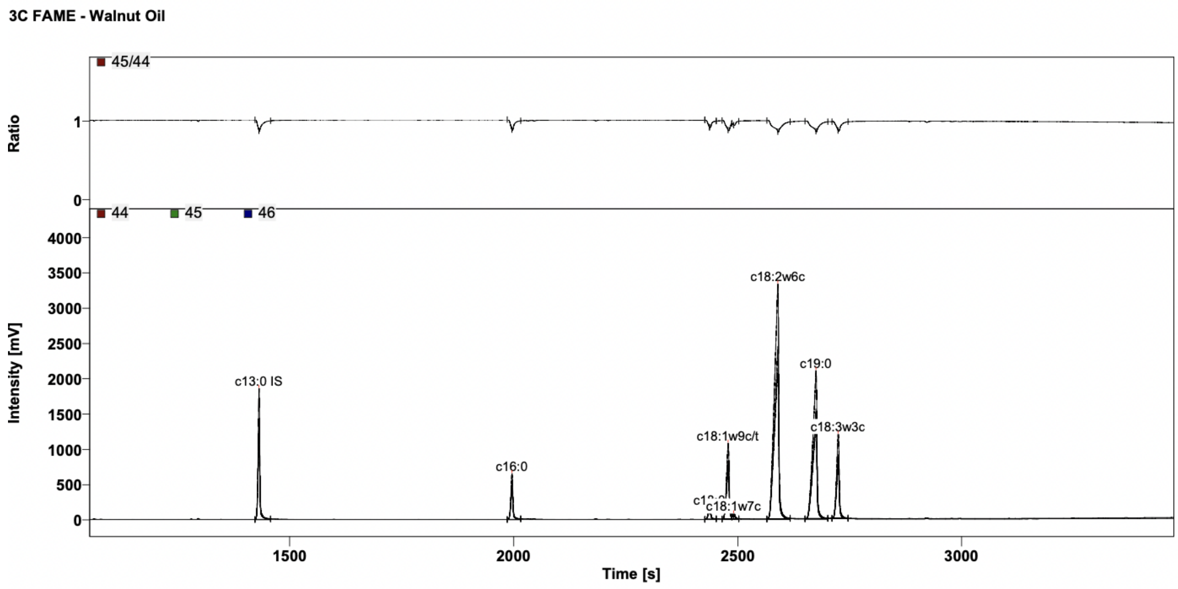

b) ^{13}C of fatty acids

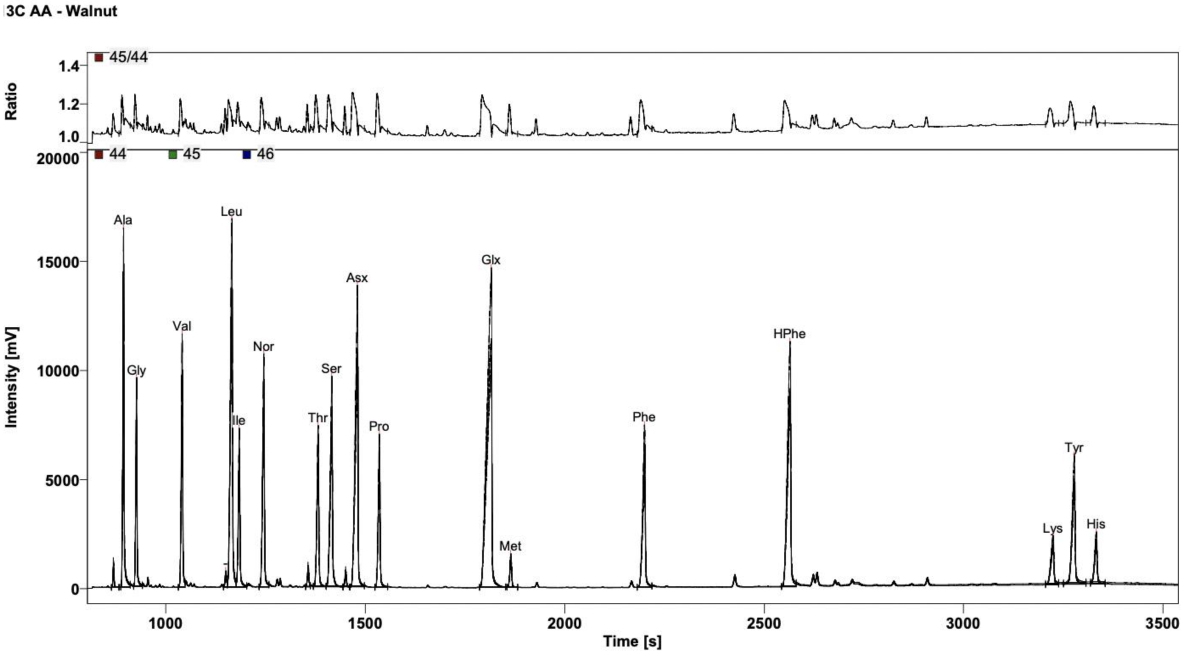

c) ^{13}C of amino acids

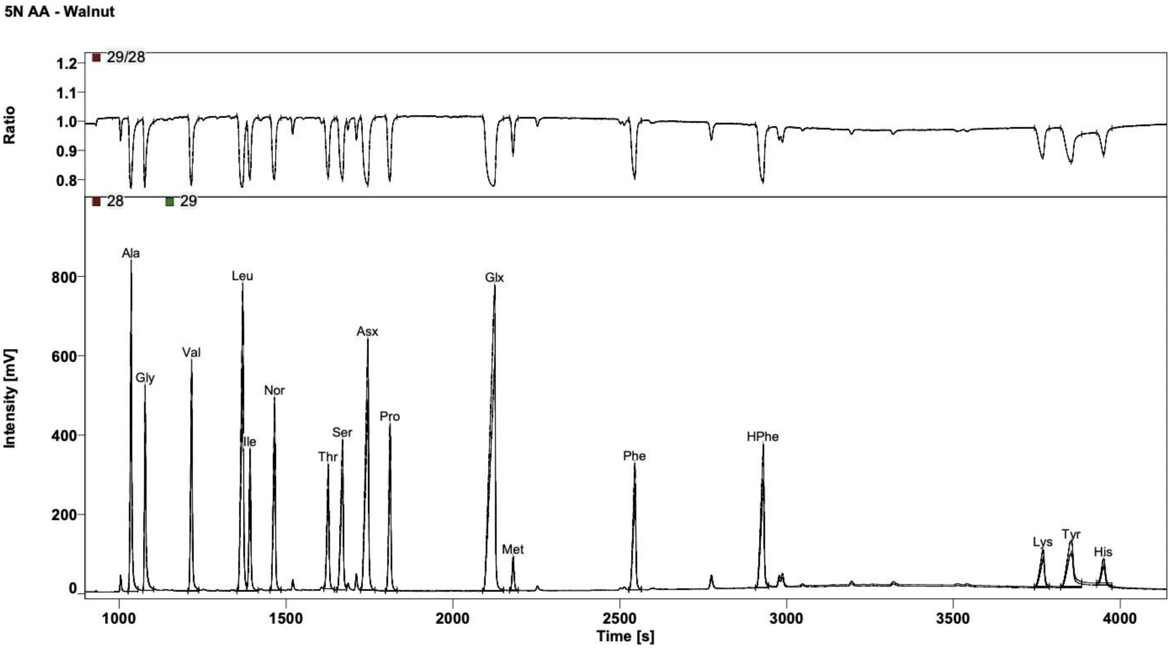

d) ^{15}N of amino acids

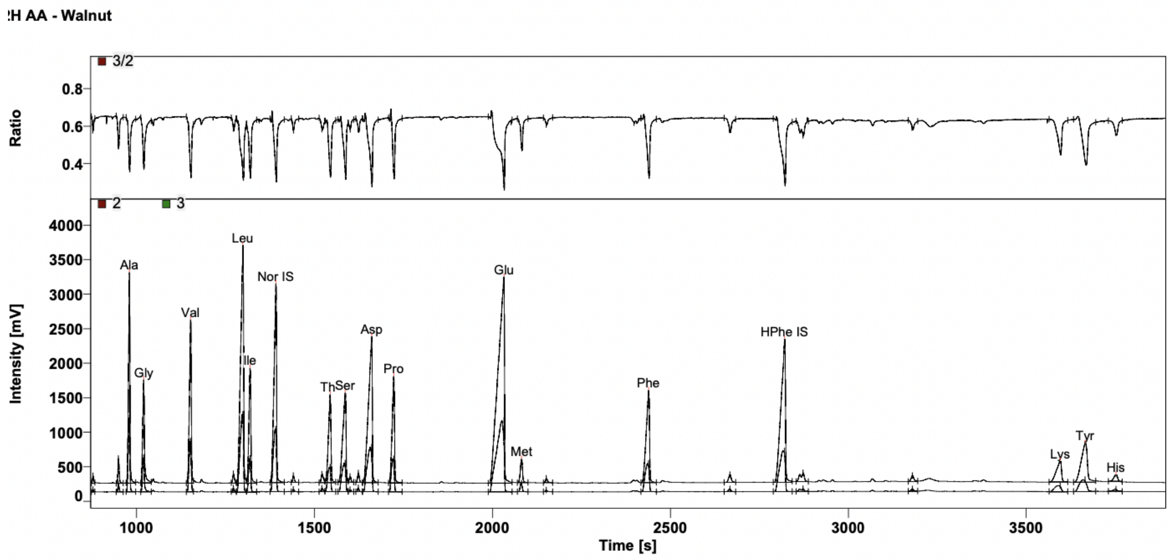

e) ^{2}H of amino acids

Figure 2. Typical chromatograms of a) ^{2}H of fatty acids, b) ^{13}C of fatty acids, c) ^{13}C of amino acids, d) ^{15}N of amino acids, and e) ^{2}H of amino acids

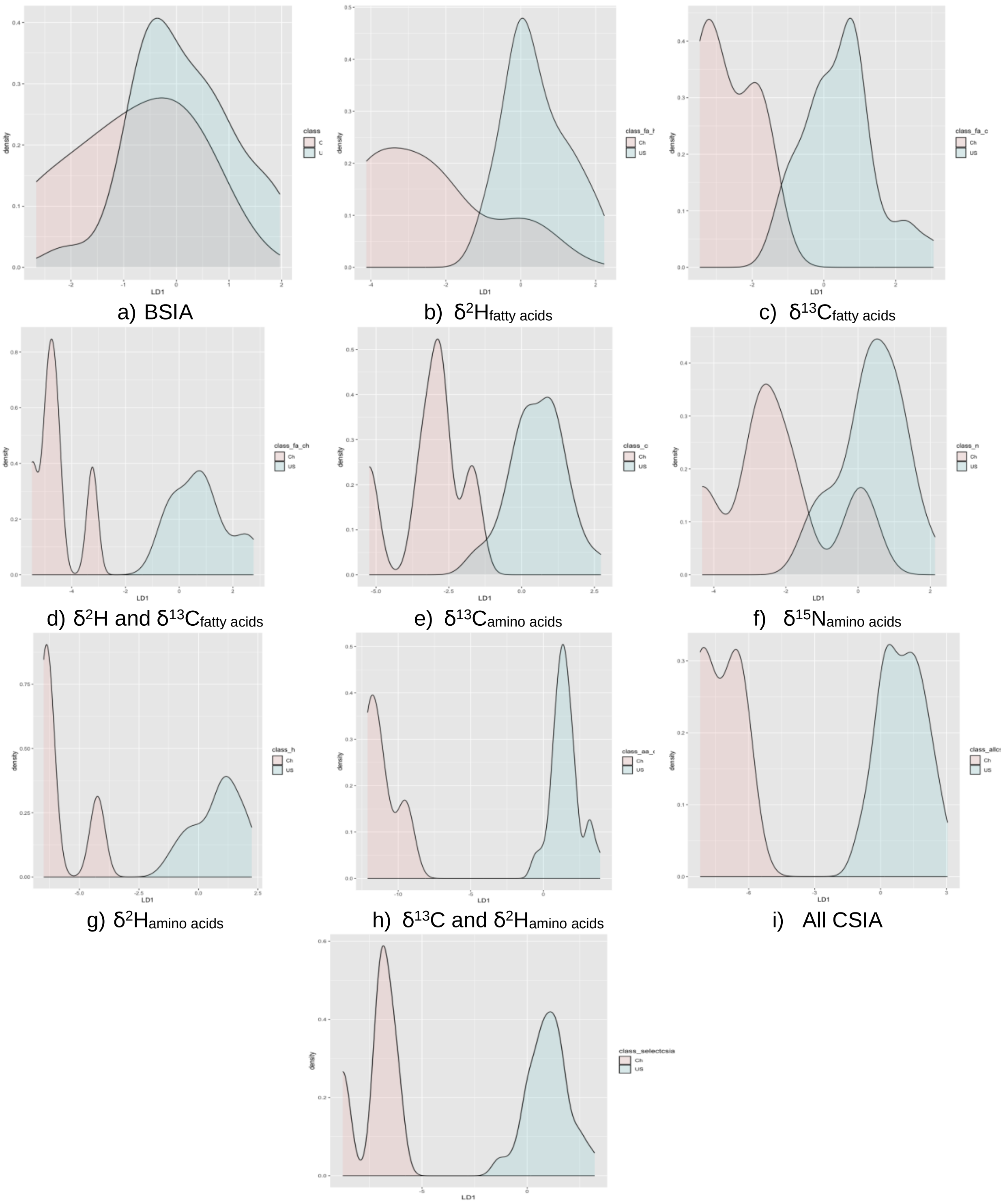

a) BSIA

b) $\delta^{2}H_{\text{fatty acids}}$

c) $\delta^{13}C_{\text{fatty acids}}$

d) $\delta^{2}H$ and $\delta^{13}C_{\text{fatty acids}}$

e) $\delta^{13}C_{\text{amino acids}}$

f) $\delta^{15}N_{\text{amino acids}}$

g) $\delta^{2}H_{\text{amino acids}}$

h) $\delta^{13}C$ and $\delta^{2}H_{\text{amino acids}}$

i) All CSIA

j) $\delta^{2}H_{C18:1\textit{n-9}}$, $\delta^{13}C_{C18:2\textit{n-6}}$, $\delta^{13}C_{C18:3\textit{n-3}}$, $\delta^{13}C_{Gly}$, $\delta^{13}C_{Leu}$, $\delta^{13}C_{Val}$, $\delta^{2}H_{Glu}$, $\delta^{2}H_{Ile}$, $\delta^{2}H_{Leu}$, and $\delta^{2}H_{Thr}$

Figure 3. LDA density plots of multiple parameters: a) BSIA, b) $\delta^{2}H_{\text{fatty acids}}$, c) $\delta^{13}C_{\text{fatty acids}}$, d) $\delta^{2}H$ and $\delta^{13}C_{\text{fatty acids}}$, e) $\delta^{13}C_{\text{amino acids}}$, f) $\delta^{15}N_{\text{amino acids}}$, g) $\delta^{2}H_{\text{amino acids}}$, h) $\delta^{13}C$ and $\delta^{2}H_{\text{amino acids}}$, i) all CSIA, and j) $^{2}H_{C18:1\textit{n-9}}$, $\delta^{13}C_{C18:2\textit{n-6}}$, $\delta^{13}C_{C18:3\textit{n-3}}$, $\delta^{13}C_{Gly}$, $\delta^{13}C_{Leu}$, $\delta^{13}C_{Val}$, $\delta^{2}H_{Glu}$, $\delta^{2}H_{Ile}$, $\delta^{2}H_{Leu}$, and $\delta^{2}H_{Thr}$ of samples sourced from China and the USA (US)
